# Transition from Target to Gaze Coding in Primate Frontal Eye Field during Memory Delay and Memory–Motor Transformation^1^,^2^,^3^

**DOI:** 10.1523/ENEURO.0040-16.2016

**Published:** 2016-04-13

**Authors:** Amirsaman Sajad, Morteza Sadeh, Xiaogang Yan, Hongying Wang, John Douglas Crawford

**Affiliations:** 1 Centre for Vision Research, York University, Toronto, Ontario M3J 1P3, Canada; 2 Neuroscience Graduate Diploma Program, York University, Toronto, Ontario M3J 1P3, Canada; 3 Department of Biology, York University, Toronto, Ontario M3J 1P3, Canada; 4 School of Kinesiology and Health Sciences, York University, Toronto, Ontario M3J 1P3, Canada; 5 Department of Psychology, York University, Toronto, Ontario M3J 1P3, Canada

**Keywords:** monkey, prefrontal, response field, sensorimotor, single-unit, spatial working memory

## Abstract

The frontal eye fields (FEFs) participate in both working memory and sensorimotor transformations for saccades, but their role in integrating these functions through time remains unclear. Here, we tracked FEF spatial codes through time using a novel analytic method applied to the classic memory-delay saccade task. Three-dimensional recordings of head-unrestrained gaze shifts were made in two monkeys trained to make gaze shifts toward briefly flashed targets after a variable delay (450-1500 ms). A preliminary analysis of visual and motor response fields in 74 FEF neurons eliminated most potential models for spatial coding at the neuron population level, as in our previous study (Sajad et al., 2015). We then focused on the spatiotemporal transition from an eye-centered target code (*T*; preferred in the visual response) to an eye-centered intended gaze position code (*G*; preferred in the movement response) during the memory delay interval. We treated neural population codes as a continuous spatiotemporal variable by dividing the space spanning *T* and *G* into intermediate *T*–*G* models and dividing the task into discrete steps through time. We found that FEF delay activity, especially in visuomovement cells, progressively transitions from *T* through intermediate *T*–*G* codes that approach, but do not reach, *G*. This was followed by a final discrete transition from these intermediate *T*–*G* delay codes to a “pure” *G* code in movement cells without delay activity. These results demonstrate that FEF activity undergoes a series of sensory–memory–motor transformations, including a dynamically evolving spatial memory signal and an imperfect memory-to-motor transformation.

## Significance Statement

Gaze-related signals in frontal cortex are often used as an experimental model for visual working memory. However, the spatial codes used during the delay between target-related visual activity and intended gaze-related motor activity remain unknown. Here, we show that frontal eye field delay activity (particularly in visuomovement neurons) shows a progressive transition through intermediate target-gaze codes, with a further jump to coding the intended gaze position in movement neurons with no delay response. Since our analytic method is based on fitting neural activity against variable behavioral errors, this suggests that such errors accumulate during the memory delay, and further escalate during the final memory-to-motor transformation. Any of these vulnerable processes might be further degraded by diseases that affect frontal cortex.

## Introduction

Primates routinely use remembered stimuli to guide spatial behavior, with varying degrees of spatial precision (Gnadt et al., 1991; White et al., 1994). This could involve a sensory-to-memory transformation, maintenance of the target in working memory, and a memory-to-motor transformation (Goldman-Rakic, 1987; Postle, 2006; Bays et al., 2011; Chatham and Badre, 2015). However, it is not known at what point in this sequence the spatial code for the sensory stimulus is transformed into a spatial code for movement, and likewise, when and how spatial errors in behavior arise (Gnadt et al., 1991; Stanford and Sparks, 1994; Krappmann, 1998; Opris et al., 2003; Faisal et al., 2008).

Memory-guided saccades provide an ideal experimental model for this question because many saccade-related neurons in the brainstem and cortex exhibit spatially selective visual, memory, and/or movement responses (Bruce and Goldberg, 1985; Funahashi et al., 1989; Wurtz et al., 2001; Schall, 2015). Further, the gaze control system, which normally controls both eye and head motion, provides convenient parameters for spatial coding (i.e., target, gaze, eye, and head) in various egocentric frames (eyes, head, or body; Freedman and Sparks, 1997; Martinez-Trujillo et al., 2003; Sajad et al., 2015). Still, a complete description of the spatiotemporal transformations in the sensory–memory–motor transformation for gaze control remains elusive.

Neurophysiological studies often trained monkeys to look toward a location that is spatially incongruent with the visual stimulus in order to dissociate target (*T*) coding in visual responses versus intended gaze position (*G*) coding in motor responses, without addressing the intervening memory delay (Gottlieb and Goldberg, 1999; Everling and Munoz, 2000; Sato and Schall, 2003). Most studies that explored this issue during delay activity used similar tasks to look for a discrete target-to-gaze switch (Funahashi et al., 1993; Mazzoni et al., 1996; Zhang and Barash, 2004). Other studies showed a gradual rotation of the population direction vector from the stimulus toward the instructed movement direction in dorsolateral prefrontal cortex (dlPFC), or a more abrupt rotation in the mediodorsal thalamus (Takeda and Funahashi, 2004; Watanabe et al., 2009). However, no previous experiment tested whether delay activity evolves across time through intermediate spatial codes (i.e., between *T* and *G*) in the visual–memory–motor transformations for saccades toward remembered stimuli.

**Figure 1.**
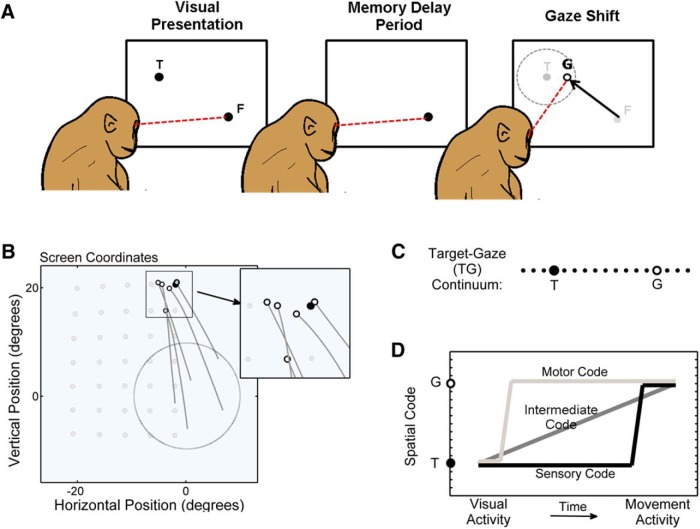
An overview of the experimental paradigm and a conceptual schematic of the possible coding schemes in the FEF. ***A***, Activity was recorded from single neurons in the FEF while monkeys performed a memory-guided gaze task with the head free to move. Monkeys initially fixated a visual stimulus (black dot labeled F) for 400-500 ms. A visual stimulus (black dot labeled *T*) was then briefly flashed on the screen for 80-100 ms (left). After an instructed delay (variable in duration; 450-850 or 700-1500 ms), the animal made a gaze shift to the remembered location of the target (gray dot labeled *T*) upon the presentation of the Go-signal. The Go-signal was the disappearance of the initial fixation target (gray dot labeled F). Inaccuracies in behavior were tolerated such that if the final gaze landed within a window around the target, a juice reward was provided. ***B***, Five gaze trajectories to a single target (black circle) within a wide array of targets (5 × 7 for this example session; gray dots) within the approximate RF location of the neuron are shown. Initial fixation positions (tail of the trajectory) were randomly varied within a central zone (large gray circle) on a trial-by-trial basis. Final gaze positions (white circles) fell at variable positions around the target. Variability in initial and final positions (relative to different frames of reference) of target, gaze (i.e., eye in space), eye (in head), and head was used to spatially differentiate sensory and various motor parameters in various frames of reference. We exploited the variability in behavioral errors to differentiate between spatial models based on target position (*T*) and final gaze position (*G*). ***C***, Additionally, a continuum of intermediary spatial models spanning *T* and *G* were constructed to treat the spatial code as a continuous variable; this allowed us to trace changes in the spatial code as activity evolved from vision to memory delay, during memory delay, and from memory delay to motor. ***D*** shows some plausible schemes for the spatiotemporal evolution of a neuronal code based on the following proposed theories: (1) the target code could be transformed into a gaze code early on, and this gaze code maintained during memory (motor theory; light gray line); (2) the target code could be maintained in the memory (sensory theory; black line) and subsequently transformed into a gaze code just before movement initiation; or (3) the spatial code could gradually change from a target code to a gaze code (dark gray line).

Assuming that one could track such codes through time, there are several ways that a *T*–*G* transition could occur in memory-guided saccades (Fig. 1*D*). A sustained *T* code followed by a late *T*–*G* transition would be compatible with sensory theories of working memory (Funahashi et al.,1993; Constantinidis et al., 2001), whereas an early *T*–*G* transition would be compatible with motor theories of working memory (Gnadt and Andersen, 1988; Gaymard et al., 1999; Rainer et al., 1999; Curtis and D'Esposito, 2006). Alternatively, *T*–*G* transition could progressively accumulate during the delay (Gnadt et al., 1991; Wimmer et al., 2014). Another possibility (data not shown) is that there is no transition of coding within any given population of cells, but rather a temporal transition of activity from a *T*-tuned population of neurons to a *G*-tuned population (Takeda and Funahashi, 2007).

The monkey frontal eye fields (FEFs), located in prefrontal cortex, are an ideal location to study this question because they are directly involved in the sensorimotor transformation for saccades and head-unrestrained gaze shifts (Bruce and Goldberg, 1985; Schall, 2015), and are part of the working memory network (Funahashi et al., 1989; O'Sullivan et al., 1995; Dias and Segraves, 1999; Sommer and Wurtz, 2001). In a recent study, we exploited the variable behavior of head-unrestrained gaze shifts to show that FEF visual and motor responses encode *T* and *G*, respectively (both relative to initial eye orientation), in saccades made toward remembered visual stimuli (Sajad et al., 2015). However, this previous analysis could not show when or how this transition happens and did not explore the contributions of individual cell types. Here, we used a similar approach, but applied our analysis in steps through time to fit a continuum of intermediate *T*–*G* models through the entire course of a memory-guided saccade task. Since this method is based on fitting spatial models against variable behavior such as errors in final gaze direction (Keith et al., 2009; Sajad et al., 2015), this also provided a direct measure of how such errors accumulate through different phases of a memory-guided gaze shift. Further, with the use of a larger dataset, we were able to categorize our cells into different memory-related (or non-memory-related) populations in order to understand their differential contributions through time to the *T*–*G* transition.

## Materials and Methods

### Surgical procedures, identification of FEF, and behavioral data recordings

**Figure 2.**
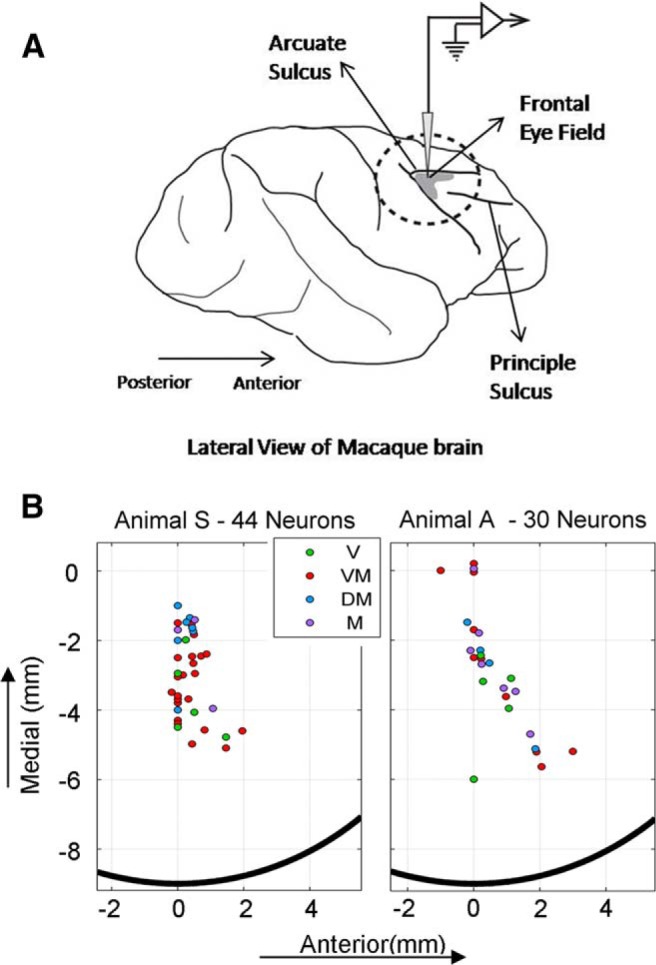
Approximate location of the FEF and the recorded sites in the two monkeys. ***A*** shows the anatomical location of the FEF, located at the anterior bank of the arcuate sulcus. ***B***, Sites within the FEF from which neurons were recorded in each animal are plotted (circles) in the coordinates of the recording chamber with the center (0,0) approximately located at the stereotaxic coordinates corresponding to the FEF (see Materials and Methods). The black semicircle represents the edge of the recording chamber. The color code represents the neuron type recorded from each site. Low-threshold microstimulation at these sites evoked saccades ranging from 2° (at the most lateral sites) and 25° (at the most medial sites) in head-restrained conditions (Bruce and Goldberg, 1985).

All protocols were in accordance with the Canadian Council on Animal Care guidelines on the use of laboratory animals and approved by the York University Animal Care Committee. The data were collected from two female *Macaca mulatta* monkeys (monkeys A and S). Each animal underwent surgeries for implanting the recording chamber (19 mm diameter), which was centered in stereotaxic coordinates at 25 mm anterior for both monkeys, and 19 mm lateral for one monkey and 20 mm lateral for the other. A recording chamber was attached over the trephination with dental acrylic (Fig. 2). In order to eliminate nonviable spatial models of neural coding from our analysis (see below), we needed to record head-unrestrained three dimensional (3-D) gaze shifts. To do this, two 5-mm-diameter sclera search coils were implanted in one eye of each animal, and two orthogonal coils were mounted on the head (Crawford et al., 1999).

### Behavioral paradigm

Monkeys were trained to perform the classic memory-guided gaze task in completely head-unrestrained conditions (Fig. 1*A*). After fixating a visual stimulus presented on the screen, a second visual stimulus (target) briefly flashed for 80-100 ms in the periphery, cuing the gaze shift goal. However, the animal had to withhold its gaze until the instruction to make a gaze shift (Go-signal = disappearance of the fixation target) was provided, at which time a gaze shift was made to the remembered location of the target. The Go-signal was presented at a random time within a flat distribution that ranged from 450 to 850 ms (for 56 of 74 neurons) or from 700 to 1500 ms (for 18 of 74 neurons). Animals were allowed a relatively large reward window of 5-12° in radius (visual angles) around the target. If the animal kept its gaze stable in the reward window for at least 200 ms after the gaze shift, a juice reward was provided. Visual stimuli were laser projected on a flat screen, which was positioned 80 cm away from the subject.

Our large reward window allowed animals to produce natural (untrained) errors in the final gaze direction (Fig. 1*B*). The variable component of these errors was necessary to dissociate the most important models (i.e., target and gaze models) described below. To quantify these, we first calculated systematic gaze errors by computing the parameters of the function [*dG* = *a*1 *dT* + *a*2], separately for vertical and horizontal components, where *dG* was gaze displacement and *dT* was target displacement from the initial gaze position. This revealed hypometria and vertical/horizontal offsets consistent with previous studies of memory-guided saccades (De Bie et al., 1987; White et al., 1994). Variable errors were quantified as the remaining errors that were unexplained by the systematic errors (i.e., residuals of the linear fit). Variable errors in behavior were distributed normally with SD in *x*-direction (SD*x*) = 6.2, and in *y*-direction (SD*y*) = 5.8 for animal S, and SD*x* = 5.9 and SD*y* = 5.7 for animal A. The average magnitude of the variable errors (mean ± SD) was 6.3 ± 6°. As we shall see, these values were sufficient to statistically separate our target and gaze models, as were other variations in 3-D eye and head orientation for the other models tested (Sajad et al., 2015).

### Extracellular recording procedures

Extracellular activity from single FEF neurons was recorded using tungsten microelectrodes (0.2-2.0 MΩ impedance; FHC). The neural signal was amplified, filtered, and stored with the Plexon MAP system for off-line cluster separation using principal component analysis with the Plexon Offline sorter software. The recorded sites were considered to be within the FEF if microstimulation with a current of <50 µA (70 ms trains of monophasic pulses; 300 µs/pulse, generated with a frequency of 300 Hz) evoked a saccade while the head was restrained (Fig. 2*B*; Monteon et al., 2010, 2012, 2013).

The search for neuron was conducted when the animal was freely scanning the environment in a lighted room with the head free to move. When a neuron with clear and stable spiking was isolated, the experiment began. A rough estimate of the receptive field (RF) of the neuron was first obtained using memory-guided gaze shifts to a wide spread of targets presented one at a time from a central fixation point. Then an array of gaze targets were set to cover the RF of the neuron, including the flanks of the RF (Fig. 1*B*, gray dots). Targets were positioned in a rectangular array (ranging between 4 × 4 and 8 × 8, 5-10° apart, depending on the size and shape of the RF). Initial fixation positions were randomized within a central window with width ranging from 10° to 40° in proportion with the estimated size of the RF (example shown in Fig. 1*B*).

### Data inclusion criteria (neurons and behavior)

We recorded neuronal activity from >200 sites in the FEFs of the two animals. However, since our method relies on a detailed analysis of the RF of single neurons, only data from sessions for which we had clear isolation of spiking data were included to eliminate any multiunit activity from analysis. Also, only neurons for which enough trials were recorded to uniformly cover a decent extent of the RF that showed either visual or presaccadic movement response types (or both) were included in the analysis. After applying our exclusion criteria, a total of 77 neurons were used for analysis (57 were previously analyzed in another study). Three of seventy-seven neurons, despite having a clear visual and/or movement response, did not exhibit any spatial tuning and thus were eliminated. So, a total of 74 neurons contributed to the results in this study. The anatomic distribution of these neurons in the recording chambers is shown in Figure 2*B*.

To obtain the behavioral data, the onset of gaze shift was defined as the time when the gaze (eye in space) velocity exceeded 50°/s, and the gaze end time was marked at the time when velocity declined below 30°/s. The final gaze positions used for spatial analysis were sampled at the gaze end time. Individual trials were excluded off-line if the gaze shift was clearly not directed toward the target or the gaze error exceeded the regression line of gaze error versus retinal error by at least 2 SDs (errors in gaze end-point scale with gaze shift size). Furthermore, trials in which the subject made an anticipatory gaze shift (with reaction time of <100 ms after the Go-signal) were eliminated to ensure that animals waited for the Go-signal (extinction of the first fixation light) to generate a saccade. In a behavioral analysis based on the same task in the same two monkeys, it was confirmed that saccade onset correlated with the Go-signal (Sadeh et al., 2015). Finally, trials in which the gaze, eye, and head were not stable during the delay period were eliminated (for details, see Sajad et al., 2015). After all trial exclusions were applied, on average, 211 trials per neuron were used for analysis.

### Neuron classification

We categorized neurons based on the temporal profile of their response (firing rate) during visual, memory, and movement periods. Note that in this experiment each trial was unique both in terms of the starting position and the metrics of the gaze shift, and a large proportion of trials were spatially spread outside of the RF hot spot, the region to which the neuron is most responsive. Therefore, in order to provide a measure of the responsiveness of a neuron, we analyzed the activity of the neuron in the 10% of trials in which the neuron was most active (Spk10), which would approximately correspond to trials that fall near the center of the best-fit RF (see next section). Spk10 was calculated for different time periods and used to identify whether a neuron had visual, delay, or movement response, as described below.

If Spk10 at 80-180 ms after target onset (an early visual period) and/or −50 to +50 ms relative to gaze onset (the perisaccadic period) was >25 spikes/s relative to the pretarget baseline, we characterized the neuron as having a visual and/or movement response (Sajad et al., 2015). A neuron was deemed responsive during the delay period if the average of the Spk10 during the 100 ms period prior to the presentation of the Go-signal was >15 spikes/s and was significantly higher than the trial-matched baseline (pretarget) activity levels (*p* < 0.05, paired-sample Wilcoxon signed rank test). These criteria resulted in a classification similar to that obtained by visual inspection, as follows: four classes including (1) visual (V) neurons, which did not exhibit movement activity; (2) visuomovement (VM) neurons, which exhibited both visual and movement responses; (3) delay-movement (DM) neurons, which did not exhibit a visual response but showed delay activity prior to the Go-signal; and (4) movement-only (M) neurons, which exhibited only a movement response starting after the Go-signal.

### Model-fitting procedures

In order to systematically test between different spatial parameters, we fit spatial models to RF data for every neuron using a procedure that has now been described several times (Keith et al., 2009; DeSouza et al., 2011; Sadeh et al., 2015; Sajad et al., 2015). In brief, the RF of the neuron was plotted by overlaying firing rate data (the number of spikes divided by the sampling window width for each trial) over two-dimensional position data corresponding to the spatial parameter related to the candidate model, such as target position relative to the eye. The predictability power of the model for the recorded data was quantified by obtaining predicted sum of squares (PRESS) residuals across all trials, which is a form of cross-validation used in regression analysis (Keith et al., 2009). Specifically, the PRESS residual for a single trial was obtained by (1) eliminating that trial from RF data, (2) fitting the remaining data points nonparametrically using Gaussian kernels at various bandwidths (2-15°), and (3) obtaining the residual between the fit and the missing data point. The overall predictability power of the model for the recorded dataset was quantified by the average of PRESS residuals across all trials for that neuron. Examples of this process will be described below. Once PRESS residuals of all tested models were obtained, the spatial code was defined as the model (using the kernel bandwidth) that yielded the overall best fit to the data.

In a preliminary analysis similar to that of our previous study (Sajad et al., 2015; which used an overlapping but smaller population of neurons), we tested all of the models that have been proposed for egocentric coding in the gaze control system against the visual and movement responses of our neurons (we did not provide allocentric visual cues, so such models were not tested). This included models of target location versus gaze, eye-in-head, and head motion (both final position and displacement) in eye-centered, head-centered, and body-centered frames of reference, for a total of 11 models (as noted above, most of these tests required the use of 3-D head-unrestrained recordings). Since this replicated our previous analysis on a smaller dataset, but with slightly better statistics, we only summarize the results here.

Target location relative to initial eye orientation (*Te*) was the best model for describing our total population of visual responses, with all other models statistically eliminated (Brown–Forsythe test). Future gaze position relative to initial eye orientation (*Ge*) gave the best overall fit for our total population of motor responses, with all other models statistically eliminated except for eye-in-head displacement and gaze displacement, which were mathematically very similar to *Ge*. Therefore, we used *Te* and *Ge* as the best representatives of visual and motor coding, abbreviated henceforth as simple *T* and *G*. Note that *G* is the visual axis in space controlled by both eye and head motion; these are still head-unrestrained data.

Note that all of these models are correlated with each other to some extent (e.g., when the target is on the right, generally gaze, eye, and head move to the right). This is why it has been so difficult to separate them using standard correlation techniques (for review, see Sajad et al. 2015). An advantage of our method is that it allows each model fit to explain all of the variations in the data that it can (even if these arise from cross-correlation), so that one then statistically compares only the data that the model cannot explain (i.e., the residuals at each point on the RF). For example, to say that *G* is statistically superior to *T* means that including errors in gaze position explains variations that cannot be accounted for by *T*, and a superior fit for *T* means that *G* errors introduce spatial variability in the fit that is not accounted for in the neural response. However, it is also possible that the ideal fit comes somewhere between *T* and *G*.

### The target–gaze continuum

**Figure 3.**
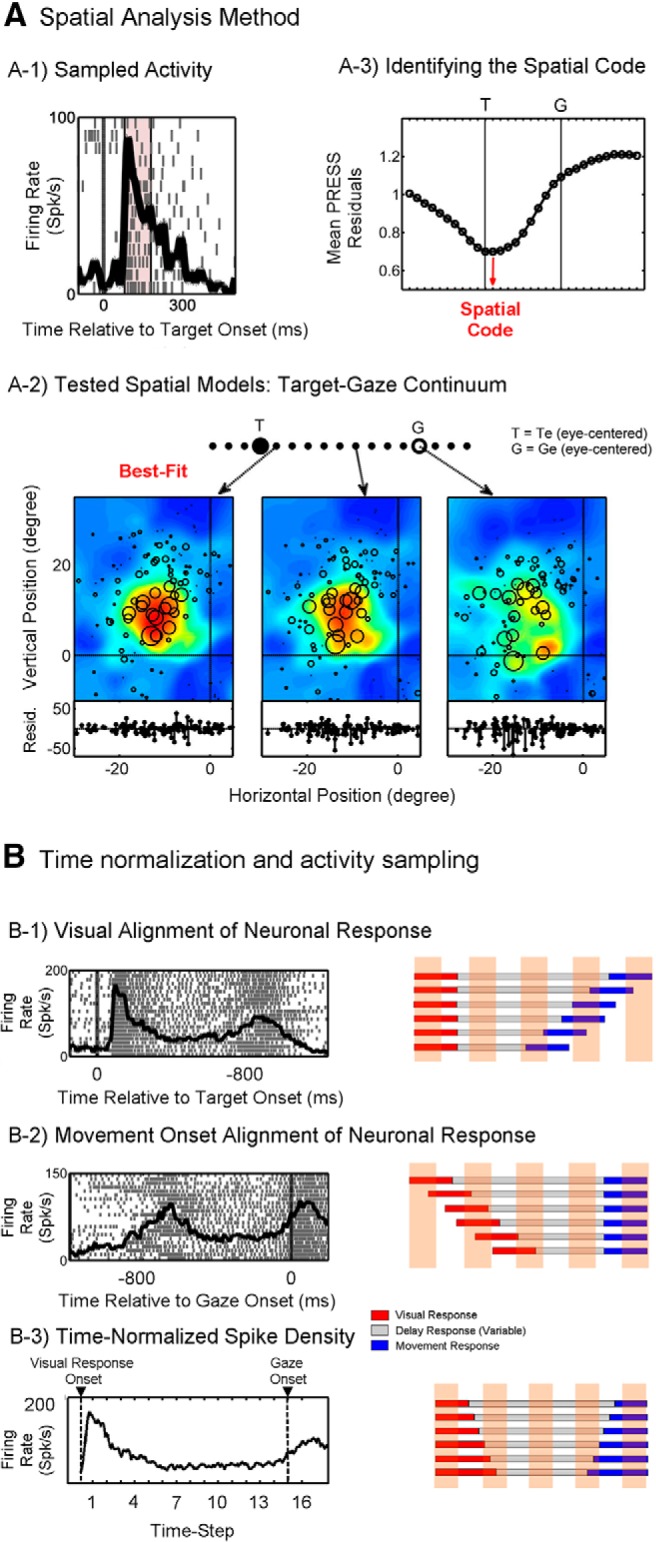
An overview of the analysis methods for identifying the spatial code and sampling neuronal activity from a time-normalized activity profile. ***A*** shows an example analysis for identifying the spatial code. ***A-1***, Here, activity from the early visual response (80-180 ms after target onset) was sampled for analysis. ***A-2*** shows the *T*–*G* continuum, and three example RF plots are shown for the visual response corresponding to the demarked models (arrows) along the *T*–*G* continuum. *T* is the eye-centered target model and *G* is the eye-centered gaze model. In the RF plots, each circle represents firing rate data (diameter) for a single trial plotted over position data corresponding to the tested model (in this study, models spanning the target model, *T*, and the gaze model, *G*). The PRESS residuals are shown at the bottom of each RF plot. In each RF plot, the color code (blue–red scale corresponding to low-to-high) represents the nonparametric fit made to all data points. ***A-3*** shows the mean PRESS (*y*-axis) as a function of tested spatial model along the *T*–*G* continuum (*x*-axis). For this example visual response the best-fit model or spatial code (lowest PRESS residuals) is the intermediate model one step away from *T* (toward *G*). Although ***A*** shows analysis only for a single sampling window, for the main analyses reported in this study we sampled activity at 16 half-overlapping time windows with the first starting at visual response onset and the last starting at gaze onset. For this, we normalized the time between visual response onset until movement onset so that we could collapse all trials together for analysis. ***B*** shows the raster and spike density plots corresponding to the classic visually aligned (***B-1***) and movement-aligned (***B-2***) neuronal responses, as well as the time-normalized spike density (***B-3***), and illustrates activity sampling based on each of these schemes.

Unlike previous studies, which only made a distinction between *T* and *G* as two possible spatial codes, we also considered intermediary codes between *T* and *G* by creating a quantitative *T*–*G* continuum between and beyond these spatial models (Fig. 1*D*). This is similar to the notion of intermediate reference frames (Avillac et al., 2005; Blohm et al., 2009; Bremner and Andersen, 2014), but here we are taking intermediate codes for two different variables within the same reference frame (eye coordinates). As described by Sajad et al. (2015), these intermediate spatial models were constructed by dividing the distance between target position and final gaze position for each trial into 10 equal intervals and 10 additional intervals extended on either tail (beyond *T* and *G*). Figure 3*A* shows an example analysis of a visual response sampled from 80 to 180 ms after target onset. The RF plots corresponding to three spatial models along the *T*–*G* continuum are shown in Figure 3*A-2*. In the RF plots, each circle represents firing rate data (diameter) for a single trial plotted over position data corresponding to the tested model (the circles are not shown in other RF plots throughout the article). The color code represents the nonparametric fit made to all data points (at a kernel bandwidth of 4°, which was the bandwidth that yielded the overall best fit for this neuron). Below each RF plot, the PRESS residuals for all data points are shown, which provide a measure for the predictability power of the model for the data points. The mean of the PRESS residuals (mean PRESS) provided the overall predictability power of the model for our dataset. Figure 3*A-3* shows the mean PRESS (*y*-axis) as a function of the tested spatial model along the *T*–*G* continuum (*x*-axis). The model that provides the lowest mean PRESS (marked by a red arrow) is the model with the highest predictability power and thus is identified as the spatial code of the neuron. For this example visual response, the best-fit model (i.e., spatial code) is the intermediate model one step away from *T* (toward *G*). Note that the RF corresponding to the best-fit model (Fig. 3*B*, left) shows a relatively high degree of spatial coherence with high neuronal response spatially confined to a restricted region (red color). The most spatially coherent fit would be a fit that gives the lowest overall variance in the data relative to each point on the RF, corresponding quantitatively to the lowest residuals of the fit. As the RF representation gets further from the best-fit representation (Fig. 3*B*, middle and right panels), the RF becomes progressively less coherent (as visualized by the size gradient of the circles and the color map), and the magnitude of the PRESS residuals increases.

### Time normalization and activity sampling for spatiotemporal analysis

The specific aims of this study required a new means of analyzing data that we have not described previously: applying our spatial analysis through discrete time steps spanning the visual, delay, and motor responses of each trial. This proved challenging because we used a variable delay period. In such a paradigm, aligning trials the standard way (with either the visual stimulus or saccade onset) results in the loss and/or mixing of activities across trials and, thus, would not allow us to trace spatial coding through the entire trial across all trials (Fig. 3*B*). To overcome this challenge, we normalized the time between an early visual period and movement onset for all trials and applied our analysis method to RFs sampled from the time-normalized activity profile. Our analytic method thus treats time and space similarly, since the spatial codes tested in this study (i.e., the *T*–*G* continuum) are also obtained through the normalization of errors in behavior (i.e., the vector difference between target position and final gaze position).

In order to sample neuronal activity using the time-normalized scheme, activity was sampled starting from an early visual period, which was the onset of the visual activity (mean, 87 ms after target onset) for visually responsive (V and VM) neurons and 80 ms after target onset for neurons with no visual response. The average (±SD) duration between this early visual period and gaze movement onset was 895 ± 234 ms across all trials. For spatiotemporal analysis, the firing rate of the neurons (in spikes per second; the number of spikes divided by the sampling interval for each trial) was sampled at 16 half-overlapping windows from these time-normalized data. This choice of sampling window numbers was based on the approximate ratio of the duration of the visual response to delay period to movement response, including a postsaccadic period starting from gaze onset (visual/delay/movement ratio is approximately 3:10:3). The final (16th) time step corresponded to an entirely postsaccadic period starting from the onset of gaze shift. Because of the time-normalization process, the sampling window width scaled with the duration between visual response onset and movement onset on a trial-by-trial basis. On the 16-step time-normalized scale, the visual burst on average lasted 2.5 steps (SD, 0.81 steps), ending by the end of the third time step in 94.5% of trials. The presaccadic duration was on average 1.35 steps (SD, 0.67), and for ∼90% of the trials started after the beginning of the 14th time step. Therefore, in the time period interleaving the first three and final three time steps, the sampled activity was largely dominated by delay activity. The average (±SD) sampling window width was 119 ± 37 ms and was no less than 50 ms for any trial, which ensured there were enough neuronal spikes captured in the sampling window to perform effective spatial analysis.

Thus, this time-normalization procedure allowed us to consider the entire sequence of visual–memory–motor responses as a continuum. It causes blurring of some other events across trials (e.g., the Go-signal) or mixing of visual and movement responses in the delay period, but these possibilities are controlled for in the Results section (see Fig. 8).

### Testing for spatial selectivity (for single neurons and population)

Our model-fitting approach would provide us with valid results if the sampled neuronal activity exhibits spatial selectivity. Therefore, we excluded data points both at the single-neuron level and at the population level that did not exhibit significant spatial tuning of any kind.

To test for spatial selectivity for a sampled response for an individual neuron, we compared the spatial selectivity of the best-fit representation with its random counterpart. To do this, we randomly shuffled the firing rate data (the number of spikes divided by the duration of the sampling window) and plotted them over the position data corresponding to the best-fit model, and repeated this procedure 100 times to obtain 100 random RFs. The PRESS residuals of these random RFs (and their respective mean PRESS values) were then obtained after fitting the data (nonparametrically, using Gaussian kernels) with the same kernel bandwidth that was used to fit the best-fit model, resulting in a total of 100 mean PRESS residuals. If the mean PRESS residuals for the best-fit model (PRESS_best-fit_) was at least 2 SDs smaller than the mean of the distribution of random mean PRESS residuals (which was normally distributed), then the sampled activity was identified as spatially selective.

At the population level, even though at a given time step some neurons exhibited spatial tuning, due to low signal-to-noise ratio or a small number of neurons contributing to the population, our estimate for the population code would not be reliable. Therefore, we excluded population data corresponding to time steps at which the mean spatial coherence of the population was not statistically higher from that of the pretarget baseline, which presumably exhibits no spatial tuning (as no task-relevant information is available). The spatial coherence for each neuron contributing to the population spatial coherence was measured using the following index:

$$\mathrm{Coherence index}=1-\left({\mathrm{PRESS}}_{\mathrm{best}-\mathrm{fit}}/{\mathrm{PRESS}}_{\mathrm{random}}\right),$$

where PRESS _random_ provided a measure of the predictability power for the random distribution (average of mean PRESS residuals over 100 independent distributions). If PRESS_best-fit_ was approximately similar to PRESS_random_, then the coherence index would be a value of ∼0. Alternatively, if PRESS_best-fit_ = 0 (which would occur only when the model perfectly accounted for the data) the index would be 1. The coherence index can also be used to determine the amount of variance in the neural data described by the best-fit model. In our data, the range of coherence indices was from −0.07 to +0.67. We did not expect the coherence index to be 1, especially because neurons in the FEF are shown to be modulated by other nonspatial factors, such as attention and reward expectancy (Schall, 2015).

### Nonparametric fits to temporal progress of spatial code in single neurons

The spatiotemporal progression of the neuronal code was analyzed by plotting the best-fit model (*y*-axis) as a function of the discretely sampled time steps (*x*-axis). To visualize these trends (and for the population analysis in the next section), we performed a nonparametric fit to these data for each neuron. Only data corresponding to spatially tuned time steps contributed to the fit. Fit values were included for every time step whose two neighboring time steps (both before and after) exhibited spatial tuning. The fit was discontinued for the range at which at least two consecutive time steps were not spatially tuned. A Gaussian kernel with a bandwidth of 1 time step was used for nonparametric fitting of these data. This choice was made conservatively to avoid oversmoothing of the data. As can be noted in Figures 5, 6, 8, 9, and 10, the fit values closely matched the data points obtained for individual neurons. Unless stated otherwise, we used the fit values, rather than individual data points, for statistical tests reported in this study, because they were less likely to be influenced by outliers.

### Population analysis and comparison between neuronal subpopulations

**Figure 4.**
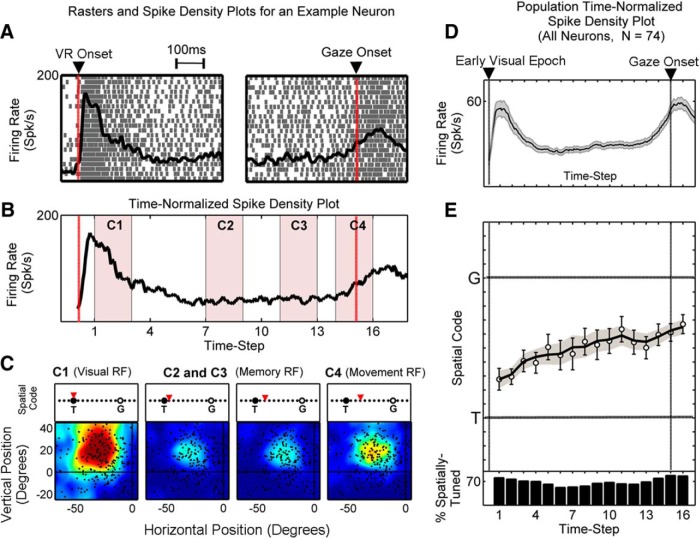
A representative neuron with visual, delay, and movement responses, and results for the overall population. ***A*** shows the visually aligned (left) and movement-aligned (right) raster and spike density plots for a VM neuron with sustained delay activity. The visual response of this neuron is from 65 to 300 ms after target onset, and the movement response begins 30 ms before gaze onset. ***B*** shows the time-normalized activity profile corresponding to ***A*** with the period between visual response (VR) onset and gaze movement onset normalized for all trials. ***C*** shows the RF maps for four time steps (***C1-C4***) sampled from the time-normalized activity profile (***B***, pink shades) with the blue-to-red color gradient representing low-to-high neuronal activity levels. The best-fit model (i.e., spatial code) at each of these time steps is depicted by a red triangle placed on the *T*–*G* continuum (panels above the RF plots). For this neuron, there was a progressive but partial shift (3 of 10 steps) in spatial code toward *G*. ***D*** depicts the time-normalized spike density for the entire population (*n =* 74), including neurons with either visual or movement response, or both. Neurons with movement-related activity beginning at or after gaze onset are eliminated. ***E*** shows the mean (±SEM) of spatially tuned best-fits at 16 half-overlapping time steps from an early visual period (visual response onset for visually responsive neurons, and 80 ms after target onset if the neuron was not visually responsive) until gaze movement-onset time. The solid line shows the mean of the fits made to individual neuron data highlighting the change in the population spatial code along *T*–*G* continuum as activity progresses from vision to movement. The histogram in the bottom panel shows the percentage of neurons that exhibited spatial tuning (*y*-axis) at a given time step (*x*-axis).

**Figure 5.**
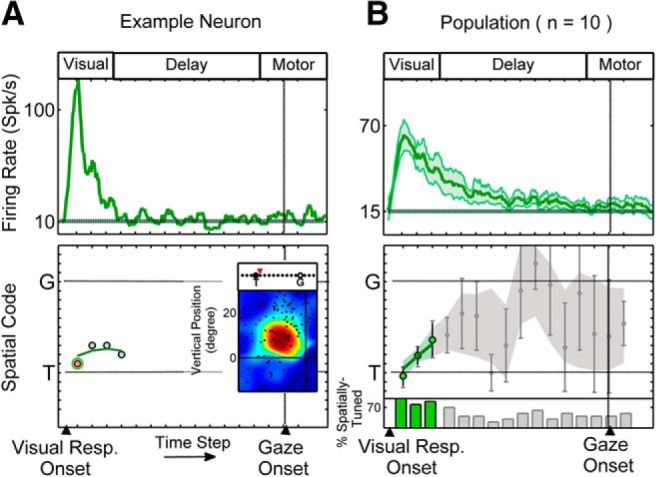
Single-neuron example and population results for V neurons. ***A*** shows the time-normalized spike density profile for an example V neuron (top) and the data points corresponding to the spatially tuned time steps across 16 half-overlapping time steps (bottom). The RF plot corresponding to the highlighted time step (bottom panel, light red circle with green boarders; first time-step here) is shown with the spatial code highlighted above the plot. ***B*** shows the population time-normalized post-stimulus time histogram (mean ± SEM) and the mean (±SEM) of the spatially tuned data points at these time steps across the V population. Colored data points (bottom) correspond to time steps at which the population spatial coherence was significantly higher than the pretarget baseline and gray shades correspond to eliminated time steps, with spatial coherence indistinguishable from pretarget baseline. The histogram shows the percentage of neurons at each time step that exhibited spatial tuning. The baseline firing rate is calculated based on the average firing rate in the 100 ms pretarget period is shown by the solid horizontal lines in spike density plots (***A***, ***B***, top). For reference, the approximate visual, delay, and motor epochs are depicted at the top of the panels.

**Figure 6.**
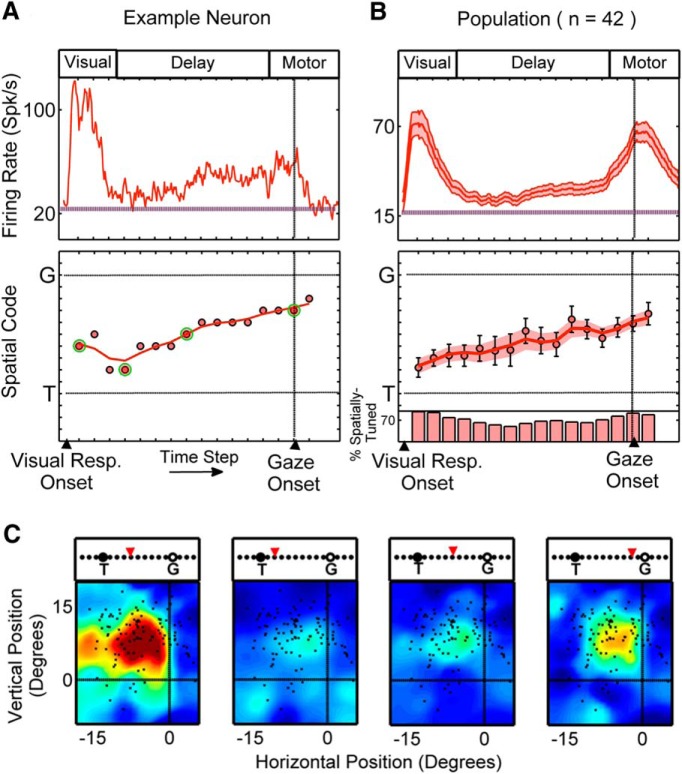
Single-neuron example and population results for VM neurons. ***A***, ***B***, Same conventions as in Figure 5. ***C***, The RF plots corresponding to time steps with highlighted data points (circles with a green border) in ***A*** (bottom) are shown, with the spatial code along the *T*–*G* continuum highlighted above each plot.

**Figure 7.**
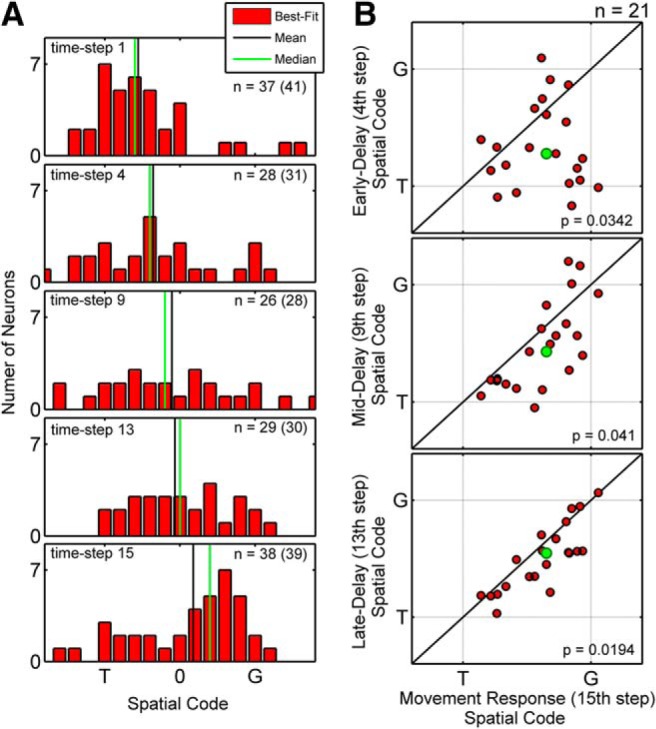
Distribution of best-fit models across the *T*–*G* continuum for the VM population through five time steps through visual, delay, and movement responses. ***A*** shows the distribution of best-fits for VM neurons for early-visual (1st time step from the time-normalized activity profile), early-delay (4th time step), mid-delay (9th time step), late-delay (13th time step), and perimovement (15th time step) intervals. Only neurons with significant spatial tuning are considered. The number of neurons contributing to each distribution is indicated on each panel (the number in brackets also includes best-fits outside of the presented range). ***B*** plots the spatial code (i.e, value of the fit to the *T*–*G* data) at each of the delay intervals (*y*-axis), versus the spatial code at the perimovement period (red dots). Here, only the 21 neurons that contributed to all five panels in ***A*** were plotted. Note the trend (from the early- to mid- to late-delay periods) for the data points to migrate toward the line of unity (i.e., toward their movement fits).

**Figure 8.**
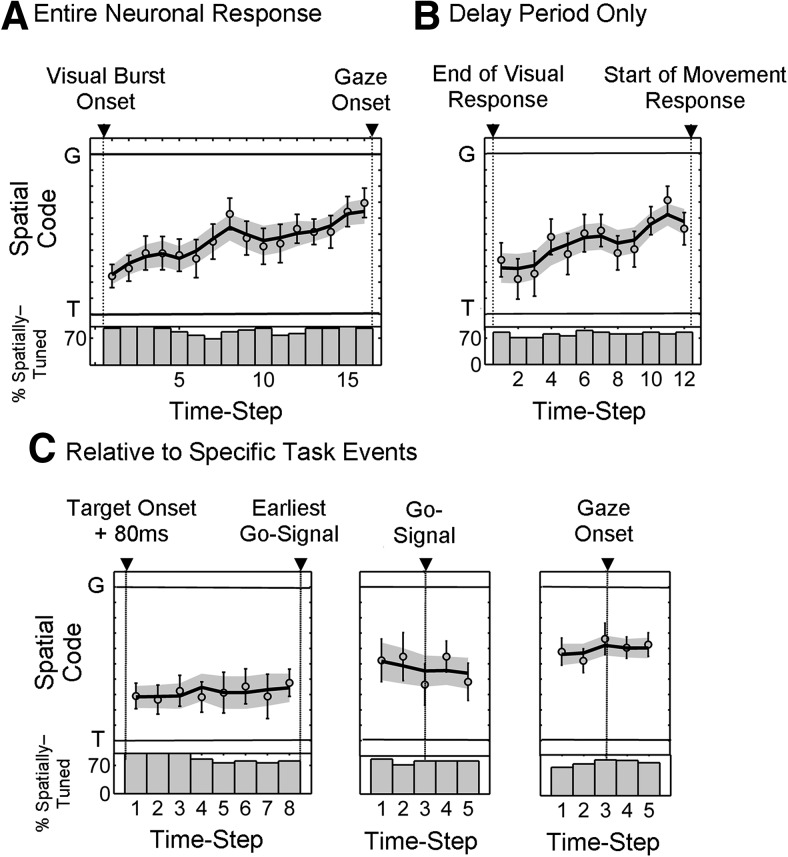
Spatiotemporal progression of neuronal code in VM neurons with sustained delay activity. ***A*** shows the results with time-normalized activity sampling, including visual and movement response using the same conventions as in Figure 5*B* (bottom). ***B*** shows the results for only the delay period, with visual and movement responses excluded. Specifically, activity was sampled from 12 half-overlapping steps from the end of the visual response (on average, 266 ms after target onset) until the beginning of the movement response (on average, 85 ms before gaze onset). This duration was on average 635 ms. ***C*** shows the spatial code at fixed times intervals relative to the following specific task events: target onset (left); the Go-signal (middle); and gaze onset (right). For target-aligned analysis (***C***, left), the time from 80 ms after target onset and the earliest Go-signal was divided into eight half-overlapping steps, resulting in a sampling window size fixed for any session but ranging between 80 and 150 ms, depending on whether the earliest Go-signal appeared at 450 or 700 ms relative to target onset for that session. The Go-signal-aligned analysis (***C***, middle) was performed using 100 ms half-overlapping windows starting at 150 ms before and extending to 150 ms after the Go-signal. The movement-aligned analysis (***C***, right) was performed using half-overlapping 100 ms sampling windows starting from 150 ms before and extending to 150 ms after gaze onset. Notice that, although there is no change in spatial code triggered by specific task events, there is a progressive change in spatial code from *T* toward *G* as we move away from the time of target presentation (left) to the time of gaze onset (right), which is in agreement with the trend seen in ***A*** and ***B***.

**Figure 9.**
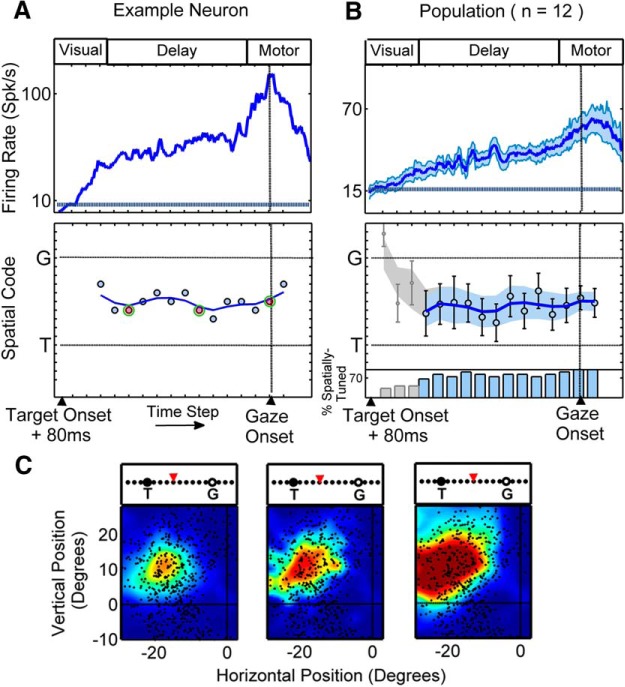
Single-neuron example and population results for DM neurons. ***A*** and ***B*** follow the same conventions as in Figure 5. ***C*** follows the same convention as in Figure 6*C*. Since these neurons lacked a visual response neuronal activity, sampling started from 80 ms after target onset.

Since most theoretical studies suggest that it is neural populations, not individual neurons, that matter most for behavior (Pouget and Snyder, 2000; Blohm et al., 2009), the results presented here focus mainly on our *T*–*G* analysis of our entire population of neurons as well as on several subpopulations (V, VM, DM, M). The overall population coding preference across the *T*–*G* continuum (Figs. 4*E*, 5*B*, 6*B*, 7, 8*B*, 9*B*, continuous trend lines) at any given time step was defined as the mean of the fits made to individual neuron data. Since the distribution of spatial code within different neuronal subpopulations did not exhibit a normal distribution, we used nonparametric statistical tests to make comparisons among data across the population, as well as the regression analyses presented in Results for VM and DM neurons.

## Results

We recorded neurons from >200 sites in the FEF during head-unrestrained conditions. After applying our rigorous data exclusion criteria, 74 neurons were included in the analysis (see Materials and Methods; Fig. 2). This is a very large number of neurons compared with other head-unrestrained studies (Freedman and Sparks 1997; Knight, 2012). However, it is not large compared with some head-restrained studies, so we limited our analysis to data that showed significant spatial tuning, and we limit our conclusions to the statistically significant neural population results described below.

As described in Materials and Methods, our preliminary data analysis corroborated the findings of the previous study (Sajad et al., 2015; i.e., that *T* provided a significantly preferred fit for the full-population visual response and future *G* provided the best overall fit for the full population motor response). We henceforth focus on the temporal transition along the *T*–*G* spatial continuum between these two events.

Figure 4*A* shows the activity profile of a typical neuron with visual, sustained delay, and movement responses using the standard conventions of aligning activity with either the onset of the visual stimulus (Fig. 4*A*, left) or the onset of the gaze shift (Fig. 4*A*, right). Figure 4*B* shows the time-normalized spike density plot corresponding to the raster and spike density plots in Figure 4*A*. The RF maps obtained at four representative time steps (C1-C4) from these data are also shown. This neuron had a very sharp (small) and spatially distinct (bound) visual RF (C1), and a similar movement RF (C4). The delay-related activity (C2, C3) exhibited similar spatial tuning, but the RF was more constricted and less spatially organized. After applying our *T*–*G* continuum analysis, we observed a progressive shift of the best-fit model from *T* part of the way toward *G* (Fig. 4*C*, red icons above the RF plots) as activity progressed in time. This trend was often observed in our preliminary analysis and thus prompted the population analyses that follow.

### Mixed-population analysis

Figure 4*D* shows the mean, time-normalized spike density profiles of the 74 neurons that qualified for our analysis (see Materials and Methods). This reveals the typical visual response (present in 52 of 74 neurons), followed by activity that was statistically significant during some or all of the delay period (present in 51 of 74 neurons), and the typical movement response (present in 64 of 74 neurons) of the FEF. For our model-fitting procedure, we sampled these data through 16 half-overlapping time steps (see Materials and Methods). The activity at each time step was first tested for spatial tuning and then the spatial code (i.e., best-fit model) was included if the result of the test was positive. At least 50% of neurons were spatially selective at each time step (Fig. 4*E*, bottom, histograms).

The mean of the individual data points at each time step (±SEM) as well as the fits made to the data points of each neuron (black line) for spatially selective responses at every time step is shown in Figure 4*E* (the median was nearly identical in this dataset; data not shown). Importantly, this method of illustrating the data (which we will use henceforth) provides the full spatiotemporal continuum of information coded by the population by showing best-fits along the *T*–*G* continuum as a function of our 16 time steps through the normalized evolution of the trials. These data reveal that the overall population best-fit model started from a location near *T* and monotonically and almost linearly moved toward *G* as activity evolved from dominantly vision related, through the delay activity, to movement related (r_s_ = 0.90, ρ = 2.44 × 10^−6^, Spearman’s ρ correlation) . On average, for the spatially tuned responses the best-fit intermediate *T*–*G* model explained 21% of the variance in the early visual activity (1st time step), while it decreased to ∼12-13% during mid-delay (7th to 9th time steps), and 23% in the perisaccadic movement period (15th time step). Since these results were better than any of the other comprehensive list of spatial models we tested, this unaccounted variance was presumably due to nonspatial factors, such as attention, motivation, and random noise.

The *T*-to-*G* progression is not due to temporal smoothing of responses between the visual–memory transition and the memory–motor transition (Fig. 3*B*), because similar trends and statistics were observed when the visual and motor responses were removed entirely from the analysis (Fig. 8, illustration of VM neurons with sustained delay activity). Framed in terms of our model-fitting method, these results mean that the population activity is initially unrelated to future gaze position errors, but, as the memory interval progresses, these variable gaze errors are increasingly reflected within the population code. Separate analysis of shorter versus longer memory intervals (data not shown) yielded no difference in the results.

To examine the contribution of different cell types to this progression in spatial coding, we subdivided our population into four subpopulations, based on whether or not they had visually evoked, delay-related, or movement-related activities (see below and Materials and Methods) and performed the same analysis for each subpopulation (Bruce and Goldberg, 1985).

### Neurons with visual responses (visual and visuomovement neurons)

Our population of neurons with visual responses was further divided into two classes based on whether or not they also exhibited movement activity (see Materials and Methods for quantitative definitions of each neuron class). In total, we had 10 V neurons and 42 VM neurons. For these neurons, activity was sampled through time from visual response onset until a postsaccadic period staring at the onset of the gaze movement, using only the epochs that tested positive for spatial tuning.

#### Visual neurons

Figure 5*A* shows the spike density profile (Fig. 5*A*, top) and model fits through time (Fig. 5*A*, bottom) for a typical V neuron, with a strong visual response but little or no delay or movement-related activity showing typical results. This neuron only exhibited spatial tuning (see Materials and Methods) at the first four time steps. The RF plot (in the best-fit representation) corresponding to the first time step, which corresponds to the early visual activity is illustrated in Figure 5*A* (bottom), showing that this visual neuron had a small and bounded RF with sharp spatial tuning. At all four time steps, the *T*–*G* continuum analysis provided fits near the *T* model (Fig. 5*A*, bottom). Most visual neurons showed a similar trend for *T* preference in the visual response, which is consistent with our previous results (Sajad et al., 2015). Figure 5*B* illustrates the corresponding analysis for the entire V neuron population, showing the mean spike density profile (Fig. 5*B*, top) and model fits through time using conventions similar to those in Figure 4, *D* and *E*. Across the V population, only the first three time steps (corresponding to the visual transient response) exhibited significantly higher spatial coherence (lower fit residuals) than the pretarget period (*p* < 0.05; green-colored data). Of the fits at these time steps (green circles), the first were very near to *T*. The next two time steps showed a trend to drift toward *G*, but none were significantly different from *T* (*p* > 0.05, one-sample Wilcoxon signed rank test). Although some V neurons showed declining activity during the delay period, this did not pass our population spatial tuning criteria (see Materials and Methods) and gave highly variable fits (gray-shaded area) that were not further considered.

#### Visuomovement neurons

A similar analysis was performed in VM neurons. VM neurons were particularly of interest in this study because they exhibited both a visual and a movement response, and, unlike V neurons, a large proportion of them exhibited delay activity (*n =* 36 of 42). Figure 6*A* (top) shows the time-normalized spike density plot for an example VM neuron with a large visual response followed by a delay response, leading to a small movement response. This neuron exhibited significant spatial tuning at all 16 time steps. The early visual response of this example was best described by intermediary models almost at the mid-point between *T* and *G*. However, from the third time step onward, there was a monotonic change in the best-fit model from a model near *T* to a model near *G* (Fig. 6*A*, bottom). RF plots corresponding to the highlighted time steps in Figure 6*A* (bottom) are shown in Figure 6*C*. Similar to the VM example shown in Figure 4*A–C*, although the RFs corresponding to the delay period are attenuated and more spatially restricted compared to the visual and movement RFs, they cover the same relative spatial position, though the spatial model that best fits each is different. The change in spatial code from *T* to *G* was present in the majority of VM neurons with delay activity: of the neurons that showed delay activity, 29 of 36 showed a positive increment along the *T*–*G* continuum. However, the degree of this change was variable across neurons (mean ± SD, 4.65 ± 6.47 *T*–*G* units).

The monotonic (constant direction) change in spatial code from *T* to *G* was also observed at the population level in the VM neurons (*n =* 42; Fig. 6*B*). Specifically, the mean population code in the first time step (corresponding to early visual response) fell close to *T* (two steps toward *G* along the *T*–*G* continuum), but, unlike V neurons, it was significantly different from *T* (*p* = 3.2 × 10^−5^, one-sample Wilcoxon signed rank test). The mean population code then progressed monotonically (almost linearly) toward *G* (R_s_ = 0.91, *p* = 9.08 × 10^−7^, Spearman’s ρ correlation). However, at the final time step (corresponding to a period within the movement response and just after gaze onset), it was still significantly different from *G* (*p* = 3.51 × 10^−7^, one-sample Wilcoxon signed rank test; Fig. 6*B*, bottom).

Figure 7*A* illustrates how the distribution of best-fits for VM neurons evolves through time. Specifically, this histogram plots the best fit *T*–*G* distributions for the early-visual (step 1), early-delay (step 4), mid-delay (step 9), late-delay (step 13), and perimovement (step 15) intervals. Focusing on the delay activity (Fig. 7*A*, middle three panels), this population did not show a bimodal distribution of *T*–*G* with a diminishing *T* peak while *G* codes rose. Instead, during the delay, spatially tuned VM neurons showed a broad distribution of *T*–*G* codes that progressively shifted toward *G* (this shift is most easily observed in the population means and medians; Fig. 7*A*, vertical black and green lines).

To visualize how this occurs at the level of individual neurons, we plotted the delay code (i.e., fits to the *T*–*G* data, see methods) as a function of the motor code for each VM neuron that showed significant spatial tuning at all five time-steps (*n* = 21). Figure 7*A* (top), corresponding to the early-delay epoch, shows that the majority of the data points were shifted below the line of unity, toward the *T* end of the distribution. Indeed, at this point in time the distribution is not significantly different from the visual distribution (0.3052, paired-sample Wilcoxon signed rank test). However, as the activity progresses through the mid-delay (Fig. 7*A*, middle) and late-delay (Fig. 7*A*, bottom) intervals, the data points progressively migrate upward, finally clustering more tightly around the motor code. At the late-delay interval, this difference is significantly different from the visual fits for the same population of neurons (*p* = 0.0190, paired-sample Wilcoxon signed rank test). When we further reduced this population to only those cells that showed significant spatial tuning at every single time step of the delay (*n* = 16), 13 of these neurons showed a positive slope in the *T*-to-*G* direction during the delay period (mean slope, 0.36 *T*–*G* units per time step; SD, 0.52 *T*–*G* units per time step).

Collectively, the results reported above support the notion that in the VM population (and in most individual VM neurons) the spatial code is not stable during the delay period but rather changes through the intermediate range between *T* and *G*, starting at a point closer to a target code and ending at a point closer to a gaze code.

To ensure that the *T*–*G* transition described above was not influenced by our time-normalization procedure, or by temporal blurring of spatial responses across different epochs, we performed a more detailed technical analysis. For this technical analysis, we used the best possible data we could obtain from our full dataset. First, we removed any VM neurons that showed any temporal discontinuity during the delay (i.e., leaving only those that showed sustained activity throughout the entire delay period; *n =* 22). Then, we repeated our time-normalized analysis (Fig. 8*A*) on these data. This yielded very similar trends and statistics to those observed for the overall population (linear progressive trend in change from a code near *T* to a code near *G*; R_s_ = 0.86, *p* = 2.40 × 10^−5^, Spearman’s ρ correlation).

Next, we performed a similar time-normalized analysis, but excluded the visual and movement responses for every neuron (Fig. 8*B*). Once again, a monotonic change in spatial code with a significant slope (R_s_ = 0.76, *p* = 0.0038, Spearman’s ρ correlation) was observed. These results show that the progressive change in the spatial code described above (Figs. 4, 6, 8*A*) is not due to the temporal smoothing of delay codes with visual and movement responses.

Finally, we controlled for the possibility that the *T*–*G* transition might have been caused by specific events within each trial, and that our time-normalization technique might have blurred these events through time to create an apparently progressive *T*–*G* transition (see Materials and Methods; Fig. 3*B*). Specifically, activity was aligned with three major task events (Fig. 8*C*), namely, target onset (Fig. 8*C*, left), Go-signal (Fig. 8*C*, middle), and movement onset (Fig. 8*C*, right). The target-aligned analysis (Fig. 8*C*, left) was performed from 80 ms after target onset until the earliest Go-signal. In this period (which was approximately equivalent for all trials for a given neuron irrespective of delay duration), the change in spatial code did not greatly contribute to the overall change in spatial code (Fig 8*C*, left). Notably, the spatial code (both the mean of the individual data points and the mean of the fits) was stable both before and after the Go-signal (Fig 8*C*, middle), suggesting that the change in spatial code was not prompted by this signal. The same observation held for gaze movement onset (Fig 8*C*, right). Collectively, these control results reinforce our main result, that the spatial code during the memory period changes progressively across the entire delay interval, rather than discretely under the influence of specific task events.

### Neurons with no visual response (delay-movement and movement-only neurons)

**Figure 10.**
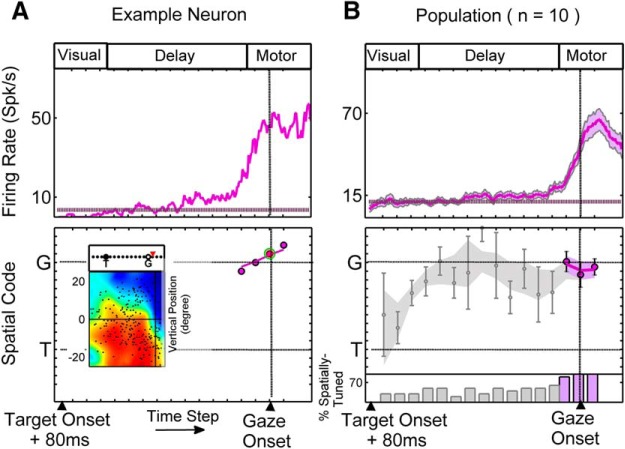
Single-neuron example and population results for M neurons. The same conventions as those in Figure 5 are used. Since these neurons lacked a visual response neuronal activity, sampling started from 80 ms after target onset.

In our population, 22 neurons exhibited movement response but lacked visual response. This movement population was further classified into the following two classes: movement neurons with activity starting at least 100 ms before the appearance of the Go-signal were classified as DM neurons (*n =* 12); and those with activity only appearing after the Go-signal were classified as M neurons (*n =* 10; see Materials and Methods). Since these neuron types lacked a visual response, the first time step used for our spatial fits (Figs. 9, 10) started from a fixed time (80 ms) after target onset.

#### Delay-movement neurons

Figure 9*A* shows the time-normalized spike density plot for a representative DM neuron, with activity beginning 150 ms after target onset, sustaining through the delay period, and leading into a presaccadic buildup toward the peak just around the time of gaze onset. This neuron first showed a spatially tuned response at the third time step. The RF plots corresponding to the 5th, 10th, and 15th (centered on gaze onset) time steps are shown in Figure 9*C*. Although there was a sudden rise in firing rate at around the time of gaze shift, there was no major change in the spatial code of this neuron through time. Instead, throughout the delay and motor epochs the spatial code of this neuron remained intermediate between *T* and *G*. At the population level, the spatial coherence of DM neurons became significantly higher than the pretarget period at the fourth time step and thereafter. At all these time steps, the spatial code remained at an intermediate position between *T* and *G*, and significantly different from both *T* (*p* = 4.88 × 10^−4^) and *G* (*p* = 0.0015), even during the movement response, just after gaze onset (i.e., final time step; one-sample Wilcoxon signed rank test) . There was no apparent trend for change in the DM fits during the delay period (Fig. 9*B*). Consistent with this, there was no significant correlation between spatial code and time step (R_s_ = 0.47, *p* = 0.20, Spearman’s ρ correlation).

#### Movement-only neurons

Figure 10*A* (top) shows the activity of an example M neuron with activity rising just before the onset of the gaze shift (∼120 ms before saccade onset). This neuron only showed spatial tuning for four time steps around the time of gaze onset, showing a spatial code tightly centered around *G* (Fig. 10*A*, bottom). The RF plot shown here corresponds to the time step centered at gaze onset. For the M population, only the three time steps straddling gaze onset showed significantly higher coherence index than the pretarget period (Fig. 10*B*, with other time steps shown in gray). In all the time steps in the motor epoch population, spatial code was very close to *G* (less than one step short of *G* along the *T*–*G* continuum) and was not significantly different from *G* (*p* > 0.25 for each time step, one-sample Wilcoxon signed rank test).

### Summary of results and comparison of subpopulations

**Figure 11.**
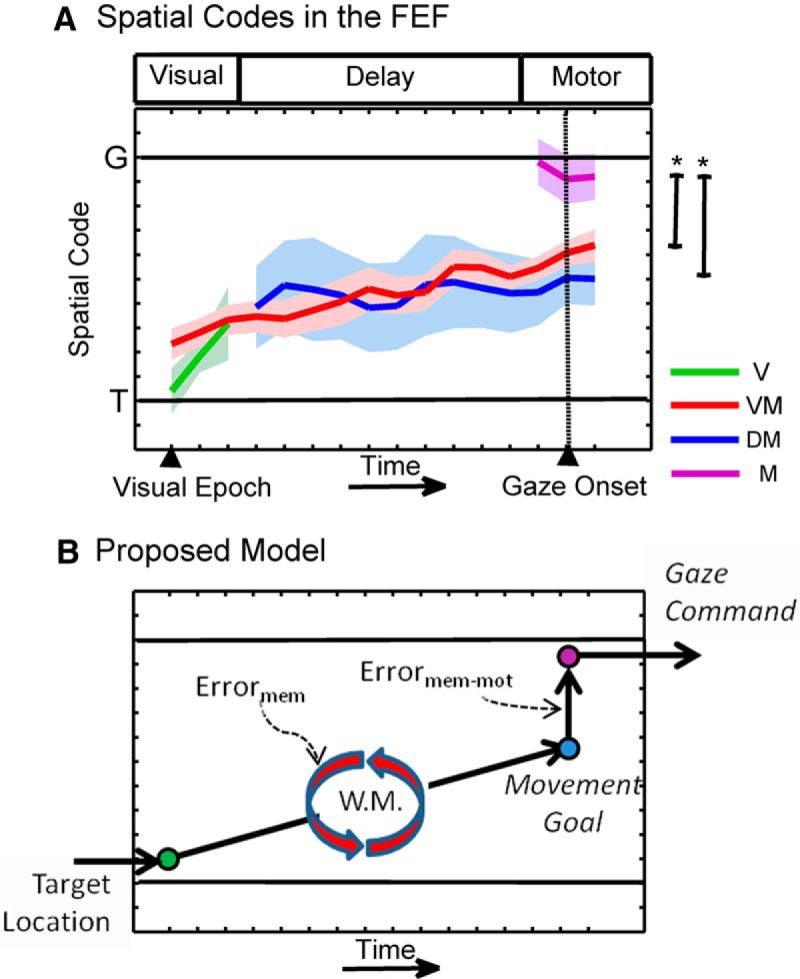
Summary of the data for different neuron types and a proposed model of the flow of spatial information within the FEF. ***A*** shows the relationship among the spatiotemporal codes of V (green), VM (red), DM (blue), and M (magenta) neurons. Asterisks (*) denote significant differences between neuron subtypes. ***B*** shows a schematic of the possible flow of information. Target location information enters the FEF (but may already have undergone some spatial processing in VM neurons). The spatial code is maintained in WM, but monotonically changes toward *G* due to memory-related (mem) processes. Upon the presentation of the Go-signal, the most recent memory of target location (i.e., movement goal) is relayed to the motor (mot) circuitry (composed of M neurons), which in turn encodes the metrics of the eminent gaze shift (*G*).

Figure 11*A* summarizes and compares the results for each of the neuron subpopulations described above, by superimposing their population means and confidence intervals within a single normalized spatiotemporal continuum plot. Based on the amount and coherence of activity in the subpopulation results described above, we have divided the neuronal responses into a visual epoch (first three time steps), the delay epoch (next 10 time steps), and the motor epoch (final three time steps, straddling gaze onset). During the visual epoch, V neurons start with a code very close to *T*, but tend to converge toward the VM code (V and VM were not significantly different in their three shared time steps). Both the VM and DM populations showed an intermediate spatial code throughout the delay period, as described above. There was no statistical difference between these two populations at any shared time steps (*p* > 0.20, two-tailed Mann–Whitney *U* test), and the slopes of the regression lines to individual data points (data not shown) were not significantly different (*p* = 0.87, linear regression comparison). However, as described above, only VM neurons showed a significant slope. The VM trend line starts closer to *T*, crosses the DM line about halfway through the delay epoch, and then ends up closer to (but still significantly different from) *G*. In summary, only VM neurons showed a significantly positive *T*–*G* slope, but all spatial coding along the *T*–*G* continuum during the visual and delay epochs (in V, VM, and DM populations) was similar, and all three would have contributed to the overall population code in these epochs.

The most striking difference between subpopulations occurs toward the end, during the motor epoch. Although three subpopulations are active at this point, only one (M) is not significantly different from *G*, and is significantly different from both the DM and VM neuron fits (*p* = 6.16 × 10^−5^ and *p* = 3.49 × 10^−5^, respectively, Bonferroni-corrected two-tailed Mann–Whitney *U* test; using data pooled across the three final time steps approximately corresponding to the motor epoch). We noted that VM neurons (but not DM neurons) showed a noticeable peak in their *T*–*G* distribution falling between the *T*–*G* midpoint and *G* (Fig. 7*A*, bottom), and wondered whether these neurons contributed more to the motor output. However, when we repeated the preceding statistical comparison, restricting the VM population to these more *G*-like codes (*n* = 27), the difference from M neurons was still significant (*p* = 0.0127, two-tailed Mann–Whitney *U* test).

To summarize, the overall impression across all four populations is of a gradual shift in coding from *T* (in the pure visual response) toward an intermediate *T*–*G* code (relayed among the V, VM, and DM populations), with a final discrete shift in coding toward *G* (i.e., a pure motor code) in the M population.

## Discussion

This is the first study to describe the entire spatiotemporal sequence of visual–memory–motor transformations during head-unrestrained gaze shifts toward remembered visual stimuli. The current study was motivated by our previous study, which used a memory-delay task to show that (1) FEF visual activity codes *T*, whereas (2) perisaccadic motor activity codes future code *G* (Sajad et al., 2015), but we did not show when or how this transition occurred. Further, we did not show how different cell populations contributed to this transition. Here, we addressed these questions by using a larger dataset (30% more neurons) and a new analytic method to track spatial coding along the *T*–*G* continuum through time. This resulted in the following two novel and important findings: (1) FEF delay activity (particularly in VM cells) showed a progressive evolution through intermediate *T*–*G* codes; and (2) an additional discrete jump occurred between intermediate *T*–*G* coding in the late delay/motor activity of VM and DM cells and *G* coding in M-only cells during the final memory–motor transformation for saccades.

Our methodology combined several advantageous approaches, as follows: (1) head-unrestrained recordings (necessary to eliminate nonrelevant spatial models in our preliminary analysis, and to provide the best behavioral estimate of frontal cortex output; Paré et al., 1994; Martinez-Trujillo et al., 2003; Corneil et al., 2007; Sajad et al, 2015); (2) a simple memory-delay saccade paradigm (avoiding the interpretive issues associated with sensory–motor dissociation tasks; Johnston et al., 2009; Hawkins et al., 2013); and (3) considering the possibility for intermediate spatial codes rather than adhering to the traditional binary classification of the spatial code as sensory or motor (the significance of this will be further elaborated on below). To our knowledge, this is the first time such a combination of techniques has been applied to the FEF or any other brain area to characterize the spatial codes in the delay period. Although head-unrestrained recordings were critical for narrowing down our analysis to *T* and *G* (and hence the intermediate *T*–*G*) models, similar results would be expected in head-restrained conditions, provided that there is enough variability in behavior to adequately separate *T* and *G*.

### Intermediary codes in the delay period

Several previous studies (Goldman-Rakic, 1987; Gnadt et al., 1991; Fuster, 2001; Postle, 2006) have proposed that spatial working memory evolves through time from a sensory to a motor code when these are dissociated in some fashion. Consistent with this, Takeda and Funahashi (2004) showed that the population spatial code in dlPFC progressively rotates from a sensory vector to a motor vector during a memory delay, in animals trained to rotate saccade direction relative to visual direction. Zhang and Barash (2004) showed a reversal from “pro” to “anti” coding across neurons in the lateral intraparietal area in the delay preceding antisaccades. In the current study, we found that FEF delay activity showed a progressive transition from a *T* code that faithfully indicated target location, through intermediate *T*–*G* codes that approached, but did not quite reach, coding a future gaze position. This *T*–*G* progression was statistically significant at the neural population level, and we observed similar trends in at least some neurons. This finding differs from the results of studies that spatially dissociated movement direction from the presented visual stimulus by virtue of cognitive manipulations (e.g., rotation or reversal) of the sensory vector (Funahashi et al., 1989, 1993; Takeda and Funahashi, 2002). In these studies, the sensorimotor transition involved a progressive decrease of activity in visually tuned cells combined with a progressive increase of activity in motor-tuned cells (Takeda and Funahashi, 2004, 2007; Zhang and Barash, 2004). We did not observe this in our simpler memory-delay task, but rather a progressive change in coding along the *T*–*G* continuum within the same population (i.e., VM neurons), even within neurons.

To our knowledge, only one other neurophysiological study has considered the change in spatial code within one population of neurons during a memory delay. Wimmer et al. (2014) found that activity in the dlPFC showed increased correlations with variations in final gaze position during a memory-delay period. Since the *T*–*G* transition observed in our results signifies a progressively increased correlation of FEF delay activity with gaze errors (discussed below), it resembles previous dlPFC results (Wimmer et al., 2014). Similar results in FEF and dlPFC are in agreement with their reciprocal connectivity and their close relationship in the maintenance of working memory (O'Sullivan et al., 1995; Sweeney et al., 1996; Offen et al., 2010). Note that the main source of the *T*–*G* progression within our full FEF population appeared to be VM neurons (Figs. 6–8). This trend was statistically significant in VM neurons; whereas, DM neurons did not show a statistically significant progression (Fig 9*B*). There is currently no clear consensus whether both classes of neurons contribute to the psychological phenomenon of working memory (Simon et al., 2002; Lawrence et al., 2005; Heinzle et al., 2007; Sommer and Wurtz, 2001). However, a survey of previous publications (Takeda and Funahashi, 2007; Takaura et al., 2011; Markowitz et al., 2015) suggests that DM neurons might be more closely associated with motor planning; whereas, VM neurons may be more closely associated with mnemonic functions. This notion is consistent with findings that visually responsive neurons are responsible for retaining and updating visual memory in the superior colliculus (SC; Sparks and Porter, 1983; Dash et al., 2015). Alternatively, it may be that all delay-responsive neurons in the gaze network are connected through an internal feedback loop for working memory, and influence each other’s spatiotemporal profiles (Curtis 2006; Okamoto et al., 2007; Verduzco-Flores et al., 2009).

### Transformations among sensory, memory, and motor codes

The second novel observation in this study was the demonstration of discrete changes in the spatial code toward *G*, in the transition among visual, memory, and motor signals. Some theoretical studies have considered spatial transformations throughout this sequence of events (Brown et al., 2004; Faisal et al., 2008; Ma et al., 2014), and some experimental oculomotor studies have inferred from their data that additional memory-to-motor transformations must occur after the delay period (Stanford and Sparks, 1994; Opris et al., 2003). However, to our knowledge, these transformations have never been directly identified in neural signals. Here we have relied on the presumption that transformations between functional networks are inherently noisy (Faisal et al., 2008; Ma et al., 2014; Alikhanian et al., 2015) to infer the occurrence of transformations based on discrete accumulations of variable errors. Our data suggest that spatial transformations might occur upstream from VM neurons, because they already show a slightly shifted intermediate code at the start of the visual response. As described above, further transition of the spatial code occurs during the memory delay, possibly due to degrading memory representations, but, importantly, there is an additional transition from an intermediate *T*–*G* code in VM/DM neurons to a pure *G* code in M neurons at the end of the delay period (even when only VM vs M neurons were compared, with a preference for gaze-related models). To our knowledge, this is the first direct demonstration of a memory-to-motor transformation between cells within the same structure.

### Conceptual model and sources of variable error

It is important to note that our model-fitting method relies on the relationship between variability in neural firing rate and variability in behavior. In particular, the *T*–*G* continuum reflects the degree to which neural firing rate faithfully represents target location for an idealized saccade, versus the variable errors in actual future gaze direction. Thus, the *T*–*G* scores shown in Figure 11*A* can be interpreted as reflecting the progression of gaze error coding in different neural populations through time. With this in mind, Figure 11*B* schematically summarizes the possible flow of spatial signals within the FEF during our task, and how these mechanisms might contribute to gaze variations.

According to this model, both V and VM neurons receive relatively unprocessed spatial information about the location of the visual stimulus relative to the eye, but VM neurons receive additional inputs from V (and perhaps other areas) containing errors that tend to shift the spatial code slightly further toward *G* along the *T*–*G* continuum. This spatial information is then maintained within a working memory/planning network composed of VM and possibly DM neurons, as well as their extrinsic connections (Zelinsky and Bisley, 2015). Here, the spatial representation in VM neurons shifts through intermediary *T*–*G* codes throughout the delay period, presumably through the accumulation of noise in a recurrent feedback network (Compte et al., 2000; Burak and Fiete, 2012; Wang et al., 2015). Upon the presentation of the Go-signal, the retained spatial information is then disinhibited, thus producing the motor response in VM and DM neurons. At the same time, this code is relayed to the M neurons, involving an additional transformation, pushing the final motor code almost to *G*. This is consistent with the notion of noise arising in the transformation from the memory to the motor network (Zheng and Wilson, 2002; Alikhanian et al., 2015; Avery and Krichmar, 2015). These signals could then influence behavior through projections to the brainstem (Künzle et al., 1976; Segraves, 1992). For example, we have observed similar noisy gaze-related signals in the motor responses of the SC (Sadeh et al., 2015).

Overall, these observations suggest that the noisy gaze signal that we observed in the overall motor response in our previous study (Sajad et al., 2015) is not the result of a random or general degradation of visual signals, but rather arises from different sources and different types of cells that relay different signals through different synaptic networks (Lawrence et al., 2005; Chatham and Badre, 2015; Markowitz et al., 2015). In simple terms, our data support a combination of the gradual progression model and late transformation models illustrated in Figure 1*D*.

### Behavioral and clinical implications

The noise-source model shown in Figure 11*B* could be useful for understanding and investigating behavior in both healthy and clinical populations. It is reasonable to assume that the sources of these variable errors would be vulnerable to diseases that affect frontal cortex function (Avery and Krichmar, 2015). If so, this confirms that the analysis of variable errors in the memory-delay saccade task has diagnostic value for diseases that affect frontal cortex function (Ploner et al., 1999). Further, whereas most behavioral studies interpret errors from memory delay tasks only in terms of maintenance (Oyachi and Ohtsuka, 1995; D'Esposito and Postle, 1999; Wimmer et al., 2014) or transformations (Henriques et al., 1998; Vesia et al., 2010; Dessing et al., 2012), our study confirms that both maintenance and memory-to-motor transformations must be taken into account (Gnadt et al., 1991; Avery and Krichmar, 2015). For example, numerous clinical studies have considered errors that arise in working memory maintenance (Minshew et al., 1999; Sweeney et al., 2007; Mazhari et al., 2010), but there is also evidence that errors arise in the gating of memory signals to action in patients with Parkinson's disease and schizophrenia (Ketcham et al., 2003; Rottschy et al., 2013; Avery and Krichmar, 2015). Thus, the observed errors in these patients could be interpreted as degraded states of noisy memory and memory-to-motor transformations observed here.

**Table 1.** Statistical table

	Analysis	Data structure	Statistical test	Power
a	Monotonicity test for spatiotemporal code: entire population	*y* = spatial code, *x* = time step	Spearman's ρ correlation	Rs = 0.90, *p* = 2.44 × 10^−6^
b	V population (1st time step) code vs T code	Normality in V code distribution not assumed, *n* = 10	One-sample Wilcoxon signed rank test	*p* > 0.05
c	VM population (1st time step) code vs T code	Normality in V code distribution not assumed, *n* = 41	One-sample Wilcoxon signed rank test	*p* = 3.2 × 10^−5^
d	Monotonicity test for spatiotemporal code: VM population	*y* = spatial code, *x* = time step	Spearman's ρ correlation	Rs = 0.91, *p* = 9.08 × 10^−7^
e	VM population (final time step) code vs G code	Normality in V code distribution not assumed, *n* = 40	One-sample Wilcoxon signed rank test	*p* = 3.51 × 10^−7^
f	Early-delay (time step 4) code vs visual response (time step 1) code	Normality in VM code distribution not assumed, *n* = 21	Paired-sample Wilcoxon signed rank test	*p* = 0.302
g	Late-delay (time step 13) code vs visual response (time step 1) code	Normality in VM code distribution not assumed, *n* = 21	Paired-sample Wilcoxon signed rank test	*p* = 0.0190
h	Figure 7B: early-, mid-, and late-delay (time steps 4, 9, 13) code vs movement response (time step 15) code	Normality in VM code distribution not assumed, *n* = 21	Bonferroni corrected; Wilcoxon test	*p* < 0.05 (see Fig 7B)
i	Monotonicity test for spatiotemporal code: VM neurons with sustained delay	*y* = spatial code, *x* = time step	Spearman's ρ correlation	Rs = 0.86, *p* = 2.40 × 10^−5^
j	Monotonicity test for spatiotemporal code (during delay-only period): VM neurons with sustained delay	*y* = spatial code, *x* = time step	Spearman's ρ correlation	Rs = 0.76, *p* = 0.0038
k	DM population (final time step) code vs T code	Normality in DM code distribution not assumed	One-sample Wilcoxon signed rank test	*p* = 4.88 × 10^−4^
l	DM population (final time step) code vs G code	Normality in DM code distribution not assumed	One-sample Wilcoxon signed rank test	*p* = 0.0015
m	Monotonicity test for spatiotemporal code: DM population	*y* = spatial code, *x* = time step	Spearman's ρ correlation	Rs = 0.47, *p* = 0.20
n	M population (final time steps) code vs G code	Normality in M code distribution not assumed, *n* ≤ 10	One-sample Wilcoxon signed rank test	*p* > 0.20
o	DM population vs VM population code	Normality in neither population distribution is assumed	Mann–Whitney *U* test	*p* > 0.25 for each time step
p	DM population vs VM population spatiotemporal progression	Two slopes obtained from: *y* = spatial code, *x* = time step	Linear regression comparison	*p* = 0.87
q1	VM population (motor epoch) vs M population (motor epoch) code	Normality in neither population distribution is assumed	Bonferroni-corrected Mann–Whitney *U* test	*p* = 6.16 × 10^−5^
q2	DM population (motor epoch) vs M population (motor epoch) code	Normality in neither population distribution is assumed	Bonferroni-corrected Mann–Whitney *U* test	*p* = 3.49 × 10^−5^
r	VM population (15th time step) code vs M neurons (15th time step) but only neurons with preference for G-like codes	Normality in neither population distribution is assumed	Mann–Whitney *U* test	*p* = 0.0127

## Synthesis

The decision was a result of the Reviewing Editor Christian Hansel and the peer reviewers coming together and discussing their recommendations until a consensus was reached. A fact-based synthesis statement explaining their decision and outlining what is needed to prepare a revision is listed below. The following reviewers agreed to reveal their identity: NONE

The manuscript describes the evolution of memory activity in the frontal eye fields from retinal to gaze coordinates. They find a gradual transition during the memory period from one coordinate frame to the other.This paper presents one of the most careful, elegant and thorough treatments of spatial coding in FEF so far reported.

The authors do a good job responding to the previous reviewers and their results of a gradual transformation from retinal to gaze coordinates in the FEF is convincing. I had one question that doesn't really pertain to the paper but has to do with the large constant error (upshift), and not the variable error, which is seen with memory based saccades. The latter variable error is used for the analyses in the paper. Do they find the constant error in FEF or does it occur later in the processing chain? Again, it is not necessary for the paper but I was just curious.

Comment of the editor: if you would like to respond to the last question please let us know. Otherwise, all previous concerns have been adequately addressed.
